# The relationship between sodium excretion and blood pressure, urine albumin, central retinal arteriolar equivalent

**DOI:** 10.1186/s12872-016-0369-1

**Published:** 2016-10-11

**Authors:** Feng Huang, Peng Yu, Yin Yuan, Qiaowei Li, Fan Lin, Zhonghai Gao, Falin Chen, Pengli Zhu

**Affiliations:** 1 Department of Geriatric Medicine, Fujian Provincial Hospital, Fujian Provincial Institute of Clinical Geriatrics, Provincial Clinical Medical College of Fujian Medical University, Fuzhou, 350001 China; 2 Department of Ophthalmology, Fujian Provincial Hospital, Fujian Provincial Institute of Clinical Geriatrics, the Provincial Clinical Medical College of Fujian Medical University, Fuzhou, 350001 China; 3 Clinical Laboratory Center, Fujian Provincial Hospital, Fujian Provincial Institute of Clinical Geriatrics, Provincial Clinical Medical College of Fujian Medical University, Fuzhou, 350001 China

**Keywords:** Sodium excretion, Blood pressure, Urinary albumin-creatinine ratio, Central retinal arteriolar equivalent

## Abstract

**Background:**

Many studies showed an association between dietary salt intake, blood pressure and increased CVD risk. The potential reason may be related to vascular structural and functional changes, through alterations in endothelial function. The central retinal arteriolar equivalent and urinary albumin reflected vascular endothelial dysfunction in different part of the body. The urinary sodium-creatinine ratio of causal urine specimens could represent the 24-h urinary sodium intake to estimate sodium intake.

**Methods:**

The 24-h sodium excretion was estimated by urinary sodium-creatinine ratio. Urinary albumin-creatinine ratio (UACR), reflecting renal arterial damage, was also determined. The central retinal arteriolar equivalent (CRAE) was detected by fundus photography and was further analyzed by semi-quantitative software.

**Results:**

Participants included 951 hypertensive patients with the average sodium excretion of 11.62 ± 3.01 g. The sodium excretion was significantly higher (*P* < 0.01) in the hypertensive as compared to that of the non-hypertensive participants. Prevalence of hypertension was increased with increasing sodium excretion. The sodium excretion was positively correlated with systolic blood pressure (SBP) and diastolic blood pressure (DBP), respectively (*r* = 0.20 and 0.14; *P < 0.01*). Furthermore, UACR and CRAE were significantly (*P < 0.01*) different within the sodium excretion quartiles (Q1-Q4). After adjusting the confounding variables, such as age and sex, the binary logistic regression analysis showed that sodium excretion was an independent factor of UACR and CRAE (*P* < 0.01).

**Conclusion:**

Our results suggest that sodium excretion in the hypertensive participants were higher. The high sodium excretion was related with the renal arterial damage as well as retinal arteriolar changes.

## Background

There is much evidence from epidemiological studies and animal experiments have shown that dietary sodium plays an important role in the regulation of blood pressure [1]. A positive relationship between sodium intake and blood pressure has been documented both in hypertensive and normotensive individuals [2, 3]. The INTERSALT study was an observational study showed an association between dietary salt intake and blood pressure. Of the study populations, four centres with low sodium excretion had low median blood pressures, low prevalence of hypertension, and either a decrease or only a small increase of blood pressure with age [4].

In a randomized double-blind crossover trial of salt restriction among mild hypertensive patients, with reductions in salt intake, there were significant falls in BP in all three ethnic groups including whites, blacks, and Asians [5]. Furthermore, a meta-analysis of 13 studies revealed that a higher salt intake was associated with greater risk of stroke (pooled relative risk 1.23, 95 % confidence interval 1.06 to 1.43; *P* = 0.007) and cardiovascular disease (1.14, 0.99 to 1.32; *P* = 0.07) [6]. The potential BP-independent increased CVD risk under a high salt diet may be related to vascular structural and functional changes, through alterations in endothelial function [7].

Retinal blood vessels are the only human microvessels that can be directly observed and quantitatively detected by many methods. Retinal microvascular lesion indicates the status of small vascular diseases in the body, and is an important indicator for predicting cardio-cerebrovascular complications [8]. The urinary albumin reflects endothelial dysfunction and renal vascular lesions, it is a risk factor of cardiovascular events in the general population, particularly in patients with hypertension [9, 10]. The risk of renal and cardiovascular events can be decreased after reducing albuminuria.

Several large cohort studies used 24 h urinary sodium excretion to estimate sodium intake, However, this method involved a considerable burden on subjects and it is difficult to collect complete and accurate 24-h urine samples [11, 12]. The urinary sodium-creatinine ratio of causal urine specimens may be an alternative method for estimating population mean levels of 24 h urinary sodium excretion, and were available for comparing different populations, as well [13].

In most of areas of China, sodium intake is above 12 g per person per day. Our study using urinary sodium-creatinine ratio of a single early morning urine to estimate 24-h urinary sodium excretion which can be used to evaluate sodium intake, aims to explore the association between sodium excretion, blood pressure and arterial injury in a Chinese population on high-sodium diets.

## Methods

### Study population

A cross-sectional survey using random and cluster sampling was performed from July 2011 through November 2011. The clusters were the individual administrative coastal villages in Fujian province of China, and we sought to obtain 7 sampling units in 14 villages for two specified townships. Invitations to participate in the survey were sent to 4616 subjects who were sampled from the 8947 inhabitants, aged 30 years and above. A total of 3343 subjects participated in the survey. We excluded 887 subjects from the analysis due to incomplete data (133 subjects), affliction with infectious disease (C-reactive protein level >10 mg/L; 39 subjects), took diuretics and angiotension converting enzyme inhibitors (25 subjects) and unqualified or unclear fundus photographs that affected the analysis (675 subjects). In the final analysis, only 2456 subjects were involved. This study was approved by the ethics committee of the Fujian Provincial Hospital, China. Written informed consent was obtained from all participants following a detailed description of the potential benefits and risks associated with the study.

### Data collection

The questionnaire survey covered the information including age, sex, occupation, smoking,alcohol habits, medical history (such as hypertension, coronary heart disease, heart failure, diabetes, stroke, or liver disease), drug use, and a family history of hypertension.

### Physical examination

Body mass index (BMI) was calculated as weight (kg)/height (m) ^2^. Blood pressure was measured on the right upper arm using a mercury sphygmomanometer by two trained internists with an appropriately sized cuff after 10 min in a sitting position without intaking tea or coffee in 30 min. The first appearance (phase I) and disappearance (phase V) of Korotkoff sounds were used to defined systolic and diastolic blood pressure (SBP, DBP). Three consecutive measurement were taken in 5 min intervals, and the average was used in the analysis.

### Blood and urine sampling and sample detection

Blood samples were collected after 8 h of overnight fasting to determine the plasma levels of triglyceride, total cholesterol, high-density lipoprotein cholesterol (HDL- C), low-density lipoprotein cholesterol (LDL- C), serum glucose, C-reactive protein (hs-CRP). Urine sample was collected in the morning. We did not ask the participants to change their dietary patterns during the collection so that the urine sodium excretion amount could be considered as a marker for usual intake. Spot urinary sodium (SUNa) by, Spot urinary creatinine (SUCr) and albumin in the urine were measured by ion-selective electrode, picric acid (LX20 automatic biochemical analyzer, USA), and Immunonephelometry (Dade Behring BN II specific protein analyzer, Germany), respectively.

The 24-h dietary sodium excretion was estimated by the following equations [13]

$$ 24 hUNa\ \left( mmol/ day\right)= 1. 2929\times {\left[ SUNa/ SUCr\times \left(\mathit{\hbox{-}} 2.04\times age+ 1 4.89\times weight\ (kg)+ 1 6.14\times height\ (cm)\mathit{\hbox{-}} 2244.45\right)/ 88\right]}^{0.392} $$

### Fundus photography

High-resolution fundus photography using a digital non-mydriatic camera was performed on both eyes (Topcon NW-8 and Nikon D90, Japan) with a capturing range of 45° using the optic disk as the center. The central retinal arteriolar equivalent (CRAE) was measured using the modified Knudtson-Parr-Hubbard formula [14] in the range of 0.5–1 DD from the disc margin. We used a semi-automated computer-based program (Singapore I Vessel Assessment [SIVA] version 3.0, jointly developed by Singapore National University and Singapore Eye Research Institute) for CRAE. A double-blind analysis of the photographs was performed by two professionally trained ophthalmologists, and high-quality fundus photographs were used for analysis.

### Diagnostic criteria and related definitions

According to the JNC7 [15], a systolic blood pressure of ≥140 mmHg or a diastolic blood pressure of ≥90 mmHg were defined as diagnostic indicators of hypertension. In addition, the patients with a hypertension history or those taking antihypertensive drugs were regarded as the population with hypertension. The lowest quartile of CRAE was defined as the central retinal artery narrowing [16]. The Estimated Glomerular filtration rate (eGFR) was calculated using the MDRD formula:

$$ eGFR\ \left( ml.mi{n}^{\mathit{\hbox{-}} 1}. 1. 7{3}^{\mathit{\hbox{-}} 1}.{m}^{\mathit{\hbox{-}} 2}\right)= 1 86. 3\times serum\  creatinine\ \left( mg.d{l}^{\mathit{\hbox{-}} 1}\right)\mathit{\hbox{-}} 1. 1 54\times Age\mathit{\hbox{-}} 0. 2 0 3\times \left( 0.742,\  if\  female\right) $$

Urinary albumin-creatinine ratio (UACR) was was calculated as $UACR\ \left( mg.{g}^{\mathit{\hbox{-}} 1}\right)= urinary\  albumin\ \left( mg.{L}^{\mathit{\hbox{-}} 1}\right)/ urine\  creatinine\ \left(g.{L}^{\mathit{\hbox{-}} 1}\right)$

Sodium excretion was divided into quartiles: the lowest quartile (Q1) corresponding to <9.04 g, the second quartile (Q2) corresponding to 9.04 g-10.73 g the third quartile (Q3) corresponding to 10.74 g-12.61 g, the highest quartile (Q4) corresponding to >12.61 g. There were all 614 cases in quartiles Q1, Q2, Q3, and Q4, respectively.

### Statistical analysis

All the data were analyzed by the SPSS 17.0 statistical software (SPSS, Inc., Chicago, IL, USA), with a *P* value <0.05 indicative of statistical significance.

The normally distributed data are shown as mean ± SD, while Skewed distributed variables are described by median (upper and lower quartile). Skewed distributed variables were taken as approximately normal distribution after logarithmic transformation for analysis. Differences in measurement data were using t-Test, one-way analysis of variance (ANOVA) and Wilconxon Rank Sum Test while the count data were analyzed by Chi-Square Test. Age-and sex-adjusted comparisons of UACR and CRAE according to the quartile of sodium excretion were made using analysis of covariance (ANCOVA). The sequential linear trend test was used for analyzing the intergroup relationships of the categorical data. Logistic regressions were used for the relationship analysis between sodium excretion, and UACR or CRAE, respectively. The factors such as age, BMI, CRAE(in the model of UACR), UACR(in the model of CRAE), total cholesterol, LDL-cholesterol, SBP, and DBP were adjusted for the regression. The chi-square test for trend was used for analyzing prevalence of hypertension within the quartiles.

## Results

### Characteristics of the participants

Table 1 gives the demographic and clinical characteristics of the study group. Study participants had a mean age of 51.4 ± 12.7 years, and included 914 (37.2 %) males and 1542 (62.8 %) females, more women than men participated. Among these subjects, 1049 (57.4 %) had a normal BMI, 806 (38.8 %) were overweight, and 241 (9.8 %) were obese. 1505 (61.3 %) were normotensive subjects, 951 (38.7 %) were hypertensive subject. All of the participants the mean sodium excretion, UACR and CRAE were 10.95 ± 2.72 g, 10.6 (4.7, 23.9) mg/g and 133.3 ± 11.1 μm.

**Table 1:** Characteristics of the 2456 study participants

Characteristics	Participants	Q1(*n* = 614)	Q2(*n* = 614)	Q3(*n* = 614)	Q4(*n* = 614)	*P* value
Age, y	51.4 ± 12.7	50.7 ± 12.8	51.5 ± 12.3	53.0 ± 12.1	57.4 ± 12.8	<0.001
male/ female	914/1542	299/315	232/382	231/383	152/462	<0.001
BMI, kg · m^2^						
<24	1049 (57.4 %)	370(60.3 %)	363(59.1 %)	354(57.7 %)	326(53.1 %)	<0.001
24–28	806(38.8 %)	197(32.0 %)	196(31.9 %)	198(32.2 %)	211(34.4 %)	<0.001
>28	241 (9.8 %)	47(7.7 %)	55(9.0 %)	62(10.1 %)	77(12.5 %)	<0.001
SBP, mmHg	129.9 ± 22.9	126.8 ± 22.0	126.9 ± 21.3	129.6 ± 21.7	136.4 ± 25.2	<0.001
DBP, mmHg	79.2 ± 11.9	77.7 ± 11.8	77.9 ± 11.5	79.7 ± 11.5	81.4 ± 12.4	<0.001
Blood pressure classification						
Normotension	1505 (61.3 %)	424(69.1 %)	421(68.6 %)	374(60.9 %)	286(46.6 %)	<0.001
Hypertension	951 (38.7 %)	190(30.9 %)	193(31.4 %)	240(39.1 %)	328(53.4 %)	<0.001
eGFR, ml · min^−1^ · 1.73^−1^ · m^−2^	106.4 ± 30.2	107.5 ± 29.2	106.7 ± 31.7	106.4 ± 30.7	103.8 ± 30.7	>0.05
Serum glucose, mmol/L	5.23 ± 1.15	5.31 ± 1.13	5.16 ± 1.13	5.16 ± 0.98	5.27 ± 1.41	>0.05
Triglyceride, mmol/L	0.8(0.6,1.2)	0.8(0.6,1.3)	0.8(0.6,1.2)	0.8(0.6,1.2)	0.8(0.6,1.3)	>0.05
Total cholesterol, mmol/L	4.97 ± 0.99	4.97 ± 1.02	4.93 ± 0.96	4.92 ± 1.03	5.02 ± 1.04	>0.05
HDL- cholesterol, mmol/L	1.22 ± 0.34	1.20 ± 0.32	1.21 ± 0.34	1.23 ± 0.34	1.22 ± 0.34	>0.05
LDL-cholesterol, mmol/L	2.73 ± 0.86	2.71 ± 0.90	2.74 ± 0.83	2.70 ± 0.87	2.79 ± 0.91	>0.05
Sodium excretion, g	10.95 ± 2.72	8.2 ± 2.5	15.1 ± 1.9	22.3 ± 2.5	40.6 ± 14.5	<0.001
UACR, mg/g	10.6(4.7,23.9)	6.2(2.9,12.5)	8.6(4.0,17.7)	11.9(5.8,22.8)	22.5(10.3,43.0)	<0.001
CRAE, um	133.3 ± 11.1	134.7 ± 10.9	133.4 ± 11.2	133.4 ± 10.9	131.5 ± 11.2	<0.001

continuous variables were reported as mean ± deviation (SD) or median (upper and lower quartile), and categorical variables as percentages (%)

Q1 group: Sodium excretion <9.04 g. Q2 group: Sodium excretion was 9.04 g-10.73 g. Q3 group: Sodium excretion was 10.74 g-12.61 g. Q4 group: Sodium excretion >12.61 g. *BMI*: body mass index, *SBP*: systolic blood pressure, *DBP*: diastolic blood pressure, *HDL-cholesterol*: high-density lipoprotein cholesterol, *LDL-cholesterol*: low-density lipoprotein protein cholesterol, *eGFR*: estimated glomerular filtration rate, *UACR*: urinary albumin-creatinine ratio, *CEAE*: central retinal artery diameter, *1 mmHg*: 0.133 kPa

### Sodium excretion in the hypertensive and non-hypertensive participants

The mean sodium excretion (10.52 ± 2.42 g) in the normotensive subjects were lower than those in the hypertensive subjects (11.62 ± 3.01 g). Prevalence of hypertension in the 24-h sodium excretion quartiles Q1–Q4 were 30.9, 31.4, 39.1, and 53.4 %, respectively. The chi-square test for trend showed that the prevalence of hypertension increased with the increasing 24-h sodium excretion within the quartiles,particularly in those with sodium excretion higher than 12.6 g, 55.62 % suffered from hypertension.

### Sodium excretion of participants within the group of sex, age, or others

(Table 2) Increased sodium excretion were associated with increasing age(*P* < 0.001). Sodium excretion increased as the quartiles of SBP and DBP increased (all *P* < 0.001). Increased sodium excretion were also associated with increasing BMI (*P* < 0.001). Sodium excretion were significantly (*P* < 0.05) higher within the high levels of total cholesterol or LDL-cholesterol. However, it was not significantly different within the sex, triglyceride, or serum glucose groups (*P* = 0.73, 0.53 and 0.88, respectively).

**Table 2:** Relationship between age, sex, blood pressure, body mass index, and sodium excretion

Item	Cases	Sodium excretion (g)
Age (years old)		
30–40	384	10.3 ± 2.4
40–50	689	10.7 ± 2.5
50–60	620	11.2 ± 2.7
60–70	482	11.3 ± 2.9
≥70	281	11.3 ± 3.1
*P* ^a^		<0.001
Sex		
Male	914	11.0 ± 2.7
Female	1542	10.9 ± 2.7
*P*		0.73
SBP (mmHg)		
Q1 80–112	546	10.4 ± 2.3
Q2 113–126	670	10.5 ± 2.4
Q3 127–142	568	11.2 ± 2.7
Q4 143–230	672	11.7 ± 3.1
*P* ^a^		<0.001
DBP (mmHg)		
Q1 50–69	484	10.3 ± 2.5
Q2 70–77	597	10.6 ± 2.5
Q3 78–85	686	11.0 ± 2.6
Q4 86–124	689	11.7 ± 3.0
*P* ^a^		<0.001
BMI (kg · m^−2^)		
15.7–21.3	614	10.2 ± 2.5
15.8–23.4	611	10.7 ± 2.6
23.5–25.6	617	11.2 ± 2.6
25.7–39.6	614	11.8 ± 2.9
*P* ^a^		<0.001
Triglyceride (mmol L^−1^)		
< 1.7	2169	10.9 ± 2.7
≥ 1.7	287	11.1 ± 2.9
*P*		0.53
Total cholesterol (mmol · L^−1^)		
< 5.2	1565	10.9 ± 2.6
≥ 5.2	891	11.1 ± 2.9
*P*		0.02
LDL-cholesterol (mmol · L^−1^)		
< 3.4	1981	10.9 ± 2.6
≥ 3.4	475	11.3 ± 3.0
*P*		<0.001
Serum glucose (mmol · L^−1^)		
< 6.0	2156	10.9 ± 2.7
≥ 6.0	300	11.0 ± 2.9
*P*		0.88

^a^Trend analysis

After adjusting BMI and age, the sodium excretion was positively correlated with systolic blood pressure (SBP) and diastolic blood pressure (DBP), respectively (*r* = 0.20 and 0.14; *P < 0.01*).

Table 3 showed The UACR and CRAE in sodium excretion quartiles. There were significant differences in UACR and CRAE between the groups within the sodium excretion Q1–Q4 quartiles (all *P* < 0.001). UACR values increased but CRAE values reduced with increasing sodium excretion levels within the quartiles. Analysis of covariance after the gender and age were adjusted showed that the increase of UACR and the decrease of CRAE within sodium excretion quartiles remained.

**Table 3:** The UACR, CRAE, and BaPWV in sodium excretion quartiles

Item	Q1 (*n* = 614)	Q2 (*n* = 614)	Q3 (*n* = 614)	Q4 (*n* = 614)	*P* value
UACR (mg.g^−1^)^a^	1.79 ± 1.23	2.17 ± 1.25	2.41 ± 1.21	2.95 ± 1.21	<0.001
CRAE (um)	134.77 ± 11.17	133.57 ± 11.49	133.52 ± 11.49	131.24 ± 10.83	<0.001

^a^The UACR values after logarithmic transformation. *UACR*: urinary albumin-creatinine ratio, *CEAE*: central retinal artery diameter

### Relationships between sodium excretion and UACR/CRAE

Logistic regression was used for analyzing the relationship between UACR/CRAE and sodium excretion. The factors such as age, BMI, CRAE, total cholesterol, LDL-cholesterol, SBP, and DBP were adjusted for the regression. The results showed that the probability of the UACR/CRAE abnormality was significantly (*P* < 0.01) increased as the sodium excretion group changed from Q1, Q2, Q3, to Q4 (Table 4). The probability of the UACR abnormality in the Q2, Q3, and Q4 group was 1.75, 2.01, and 5.62 times as that of the Q1 group. The probability of the CRAE abnormality was 1.22, 1.11, and 1.36 times higher in the Q2, Q3, and Q4 group as compared to that of the Q1 group. It was not significantly (*P* > 0.05) different in Q1, Q2, and Q3 groups but was significantly different between group Q1 and Q4 (*P* = 0.041).

**Table 4:** Logistic regression analysis demonstrating the relationship of UACR/CRAE and Sodium excretion

Sodium excretion group	UACR						CRAE					
	Unadjusted OR (95 % CI)	*P*	*P* ^*a*^	Adjusted OR (95 % CI)	*P*	*P* ^*a*^	Unadjusted OR (95 % CI)	*P*	*P* ^*a*^	Adjusted OR (95 % CI)	*P*	*P* ^*a*^
Q1	1.00 (control)	——	<0.001	1.00 (control)	——	<0.001	1.00 (control)	——	<0.001	1.00 (control)	——	<0.001
Q2	1.78 (1.22–2.58)	0.003		1.75 (1.18–2.59)	0.005		1.28 (0.98–1.67)	0.068		1.22 (0.92–1.61)	0.176	
Q3	2.24 (1.56–3.22)	<0.001		2.01 (1.37–2.95)	<0.001		1.18 (0.91–1.55)	0.219		1.11 (0.83–1.47)	0.489	
Q4	7.10 (5.08–9.92)	<0.001		5.62 (3.93–8.06)	<0.001		1.61 (1.24–2.09)	<0.001		1.36 (1.01–1.82)	0.041	

Adjusted variables included age, sex, BMI, CRAE, Triglyceride, Total cholesterol, HDL-cholesterol, LDL-cholesterol, Serum glucose, eGFR, SBP, and DBP

^a^Trend analysis

## Discussion

Epidemiological studies confirmed that excessive sodium intake could cause high blood pressure, and that a high sodium diet could be an important risk factor for hypertension [17]. In general, the accurate estimation of sodium content in the diet is difficult. With normal renal function, an individual can excrete 90.4–95 % of the sodium intake in the urine by the kidneys. Therefore, for an individual with stable diet, the 24-h urinary sodium excretion basically reflects the level of sodium intake without taking medication which could interfere sodium intake such as diuretics, and is also a reliable method to index the sodium intake [18, 19]. A high urinary sodium reflects a high sodium diet to some extent [13, 18]. The urinary sodium-creatinine ratio of causal urine specimens [13] and second morning voiding urine collecting [20] were correlated with the 24-h urinary sodium excretion, which could represent the 24-h urinary sodium excretion. A single urine collection is simple, easy, and suitable for the epidemiological study of a large number of samples. Hence, this study used the first morning urine samples and evaluated 24-h urinary sodium excretion to illustrate the level of sodium intake based on the urinary sodium-creatinine ratio.

Previous studies have shown that with the increase of sodium intake, SBP and DBP were also significantly increased [21]. Furthermore, it has been shown that reduction of dietary sodium intake lowered the SBP and DBP levels [22, 23]. Consistent with the previous studies, the results of this study also showed that the SBP and DBP levels were significantly correlated with sodium excretion. As far as we know, the conclusion may be explained by the phenomenon of salt sensitivity of BP, which refers to the BP responses for changes in dietary salt intake to produce meaningful BP increases or decreases. Epidemiologic data demonstrate the role of high dietary salt intake in mediating cardiovascular and renal morbidity and mortality. Recent studys suggested that salt sensitivity seem to be related not only the kidney malfunction but also the endothelial dysfunction [24].As such, it is necessary to take measures to reduce the sodium intake in the coastal areas of China in order to lower the blood pressure levels and reduce cardiovascular events in some individuals.

The typical diet may also effect sodium excretion, such as potassium: sodium ratio. The urinary potassium: sodium ratio in the INTERSALT study had a significant, inverse relation with blood pressure. This ratio bore a stronger statistical relationship to blood pressure than did either sodium or potassium excretion alone [4]. In the DASH trial, a diet rich in fruits and vegetables, which were found forms of potassium that do not contain chloride, offered larger cellular entry in exchange for sodium and greater antihypertensive effects, as compared with the typical American diet [25].

An increased urinary albumin excretion has been reported in the patients with hypertension or diabetes, as well as the individuals with a normal blood pressure [20, 26]. Microalbuminuria could reflect the renal arteriolar damage, and is closely associated with cardiovascular events and mortality [27].This study showed that with an increase in the urinary sodium excretion, the urinary albumin excretion along with the incidence of UACR abnormalities (greater than 30 mg g^−1^) were increased. After the adjustment of factors, such as age and blood pressure, the incidence of UACR abnormalities still remained higher than normal with an increase in the urinary sodium excretion. This observation indicated that a high-sodium diet was an independent risk factor for the UACR anomalies. A high sodium diet increased the urinary albumin excretion along with the risk of cardiovascular events. Similarly, Verhave et al. [28] reported that a high sodium diet exacerbated the high UACR-induced cardiovascular and renal damages. The related mechanism involved could be due to the sodium overload-induced neurohumoral reactions that could cause systolic and diastolic abnormalities of the renal afferent and efferent arterioles, which could further lead to abnormal glomerular filtration rate, vascular endothelial damage, and hemodynamic abnormalities [29].

Retinal vascular and cardiovascular systems have a common anatomical and physiological characteristic. They are the only blood vessels that can be non-invasively observed in the body, and have attracted a great attention. The population-based epidemiological survey of the eye-fundus blood vessels demonstrated that retinal vascular abnormalities could increase cardiovascular mortality [30]. These surveys included: the atherosclerosis risk in community, the cardiovascular health, the beaver dam eye, and the Blue Mountains eye studies. A prospective study [31] revealed that the retinal vascular changes might occur before the onset of the cardio-cerebrovascular disease, and could be independent predictors of the occurrence and prognosis of systemic diseases. In this study, we found that the central retinal artery diameter was decreased with increasing urinary sodium excretion. After adjustment of factors such as age, sex, and blood pressure, the logistic regression showed that the risk of the central retinal artery diameter abnormalities was still significant in the participants with high sodium excretion. It further suggested that a high sodium diet was an independent risk factor for the central retinal arterial damage, which could increase the risk of cardiovascular disease.

However, we were aware of the limitations of this study. In our cross-sectional study, the exposure and outcome variables coexisted, which could have led to difficulty in clarifying the causal relationship between sodium excretion and vascular damage. In addition, some information on the participants was obtained through questionnaires, which could have led to an inevitable information bias. And according to the including criteria, we already excluded the population who have been on long-term usage of diuretics or ACEI/ARB which may affect the UACR. However, other antihypertensive treatment was not adjusted in the multivariable-adjusted models, which could have affected our results.

## Conclusion

Our results suggest that sodium excretion in the hypertensive participants were higher. The high sodium excretion was related with the renal arterial damage as well as retinal arteriolar changes.

## Abbreviations

BMI: Body mass index
CRAE: Central retinal arteriolar equivalent
CVD: Risk factor for cardiovascular disease
DBP: Diastolic blood pressure
eGFR: Estimated glomerular filtration rate
HDL- C: High-density lipoprotein cholesterol
hs-CRP: C-reactive protein
LDL- C: Low-density lipoprotein cholesterol
SBP: Systolic blood pressure
SUCr: Spot urinary creatinine
SUNa: Spot urinary sodium
UACR: Urinary albumin-creatinine ratio

## References

[1] He FJ, MacGregor GA (2009). A comprehensive review on salt and health and current experience of worldwide salt reduction programmes. J Hum Hypertens.

[2] Elliott P, Stamler J, Nichols R (1996). Intersalt revisited: further analyses of 24 hour sodium excretion and blood pressure within and across populations. BMJ.

[3] Zhou BF, Stamler J, Dennis B (2003). Nutrient intakes of middle-aged men and women in China, Japan, United Kingdom, and United States in the late 1990s: The INTERMAP Study. J Hum Hypertens.

[4] Intersalt Cooperative Research Group (1988). Intersalt: an international study of electrolyte excretion and blood pressure. Results for 24 hour urinary sodium and potassium excretion. BMJ.

[5] He FJ, Marciniak M, Visagie E (2009). Effect of modest salt reduction on blood pressure, urinary albumin, and pulse wave velocity in white, black, and Asian mild hypertensives. Hypertension.

[6] Strazzullo P, D’Elia L, Kandala NB (2009). Salt intake, stroke, and cardiovascular disease: meta-analysis of prospective studies. BMJ.

[7] Sanders PW (2009). Vascular consequences of dietary salt intake. Am J Physiol Renal Physiol.

[8] Ikram MK, de Jong FJ, Bos MJ (2006). Retinal vessel diameters and risk of stroke: the Rotterdam Study. Neurology.

[9] Hillege HL, Fidler V, Diercks GF (2002). Urinary albumin excretion predicts cardiovascular and noncardiovascular mortality in general population. Circulation.

[10] Busari OA, Opadijo OG, Olarewaju OT (2010). Microalbuminuria and its relations with serum lipid abnormalities in adult Nigerians with newly diagnosed hypertension. Ann Afr Med.

[11] Daviglus ML, Greenland P, Stamler J (2005). Relation of nutrient intake to microalbuminuria in nondiabetic middle-aged men and women: International Population Study on Macronutrients and Blood Pressure (INTERMAP). Am J Kidney Dis.

[12] Aaron KJ, Campbell RC, Judd SE (2011). Association of dietary sodium and potassium intakes with albuminuria in normal-weight, overweight, and obese participants in the Reasons for Geographic and Racial Differences in Stroke(REGARDS) Study. Am J Clin Nutr.

[13] Tanaka T, Okamura T, Miura K (2002). A simple method to estimate populational 24-h urinary sodium and potassium excretion using a casual urine specimen. J Hum Hypertens.

[14] Knudtson MD, Lee KE, Hubbard LD (2003). Revised formulas for summarizing retinal vessel diameters. Curr Eye Res.

[15] Chobanian AV, Bakris GL, Black HR (2003). Seventh report of the joint national committee on prevention, detection, evaluation, and treatment of high blood pressure. Hypertension.

[16] Sabanayagam C, Shankar A, Klein BE (2011). Bidirectional association of retinal vessel diameters and estimated glomerular filtration rate decline: The Beaver Dam CKD Study. Am J Kidney Dis.

[17] Adrogue HJ, Madias NE (2007). Sodium and potassium in the pathogenesis of hypertension. N Engl J Med.

[18] Rose G, Stamler J (1989). The INTERSALT study: background, methods and main results. INTERSALT Co-operative Research Group. J Hum Hypertens.

[19] Chen CM, Zhao W, Yang Z (2008). The role of dietary factors in chronic disease control in China. Obes Rev.

[20] Kawasaki T, Itoh K, Uezono K (1993). A simple method for estimating 24 H urinary sodium and potassium excretion from second morning voiding urine specimen in adults. Clin Exp Pharmacol Physiol.

[21] Umesawa M, Iso H, Date C (2008). Relations between dietary sodium and potassium intakes and mortality from cardiovascular disease: the Japan Collaborative Cohort Study for Evaluation of Cancer Risks. Am J Clin Nutr.

[22] He FJ, Li J, Macgregor GA (2013). Effect of longer term modest salt reduction on blood pressure: Cochrane systematic review and meta-analysis of randomised trials. BMJ.

[23] Larsson SC, Virtanen MJ, Mars M (2008). Magnesium, calcium, potassium, and sodium intakes and risk of stroke in male smokers. Arch Intern Med.

[24] Hoon Young C, Hyeong Cheon P, Sung Kyu H (2015). Salt sensitivity and hypertension: a paradigm shift from kidney malfunction to vascular endothelial dysfunction. Electrolyte Blood Press.

[25] Appel LJ, Moore TJ, Obarzanek E (1997). A clinical trial of the effects of dietary patterns on blood pressure. DASH Collaborative Research Group. N Engl J Med.

[26] Kim BJ, Lee HJ, Sung KC (2007). Comparison of microalbuminuria in blood pressure categories of prehypertensive subjects. Circ J.

[27] Hillege HL, Fidler V, Diercks GF (2002). Urinary albumin excretion predicts cardiovascular and noncardiovascular mortality in general population. Circulation.

[28] Verhave JC, Hillege HL, Burgerhof JG (2004). Sodium intake affects urinary albumin excretion especially in overweight subjects. J Intern Med.

[29] du Cailar G, Ribstein J, Mimran A (2002). Dietary sodium and target organ damage in essential hypertension. Am J Hypertens.

[30] Wong TY, Klein R, Sharrett AR (2003). The prevalence and risk factors of retinal microvascular abnormalities in older persons: the Cardiovascular Health Study. Ophthalmology.

[31] Wong TY, Shankar A, Klein R (2004). Prospective cohort study of retinal vessel diameters and risk of hypertension. BMJ.

